# Obstructive Sleep Apnea as a Predictor of a Higher Risk of Significant Coronary Artery Disease Assessed Non-Invasively Using the Calcium Score

**DOI:** 10.3390/life13030671

**Published:** 2023-03-01

**Authors:** Piotr Macek, Monika Michałek-Zrąbkowska, Barbara Dziadkowiec-Macek, Małgorzata Poręba, Helena Martynowicz, Grzegorz Mazur, Paweł Gać, Rafał Poręba

**Affiliations:** 1 Department of Internal Medicine, Occupational Diseases, Hypertension and Clinical Oncology, Wroclaw Medical University, 213 Borowska St., 50-556 Wroclaw, Poland; 2 Department of Paralympic Sports, Wroclaw University of Health and Sport Sciences, Witelona 25a, 51-617 Wroclaw, Poland; 3 Department of Population Health, Division of Environmental Health and Occupational Medicine, Wroclaw Medical University, Mikulicza-Radeckiego 7, 50-368 Wroclaw, Poland

**Keywords:** coronary artery calcium score, coronary artery disease risk, obstructive sleep apnea

## Abstract

The aim of this study was to assess the coronary artery calcium score in patients with obstructive sleep apnea (OSA). The study group (group A) consisted of 62 patients with diagnosed obstructive sleep apnea (mean age: 59.12 ± 9.09 years, mean AHI index in polysomnography: 20.44 ± 13.22/h), and 62 people without diagnosed obstructive sleep apnea (mean age 59.50 ± 10.74 years) constituted the control group (group B). The risk of significant coronary artery disease was assessed in all patients, based on the measurement of the coronary artery calcium score (CACS) using computed tomography. The following cut-off points were used to assess the risk of significant coronary artery disease: CACS = 0—no risk, CACS 1–10—minimal risk, CACS 11–100—low risk, CACS 101–400—moderate risk, and CACS > 400—high risk. Group A was characterized by statistically significantly higher CACS than group B (550.25 ± 817.76 vs. 92.59 ± 164.56, *p* < 0.05). No risk of significant coronary artery disease was statistically significantly less frequent in group A than in group B (0.0% vs. 51.6%, *p* < 0.05). A high risk of significant coronary artery disease was statistically significantly more frequent in group A than in group B (40.3% vs. 4.8%, *p* < 0.05). In group A, patients with severe OSA and patients with moderate OSA had statistically significantly higher CACS than patients with mild OSA (910.04 ± 746.31, 833.35 ± 1129.87, 201.66 ± 192.04, *p* < 0.05). A statistically significant positive correlation was found between the AHI and CACS (r = 0.34, *p* < 0.05). The regression analysis showed that OSA, male gender, older age, type 2 diabetes, peripheral arterial disease, and smoking were independent risk factors for higher CACS values. AHI ≥ 14.9 was shown to be a predictor of a high risk of significant coronary artery disease with a sensitivity and specificity of 62.2% and 80.0%, respectively. In summary, obstructive sleep apnea should be considered an independent predictive factor of a high risk of significant coronary artery disease (based on the coronary artery calcium score).

## 1. Introduction

Coronary artery disease (CAD) is defined as the presence of atherosclerosis in coronary arteries [1]. The formation of atherosclerotic plaque is a complex process that depends on many factors affecting the intimal layer of the coronary artery wall. Endothelial dysfunction, oxidized serum lipids, inflammation, and thrombosis, with secondary effects of angiogenesis and calcification, are involved in the pathogenesis of plaque formation and progression [2]. Coronary heart disease includes the diagnosis of stable angina, acute coronary syndrome, and silent myocardial ischemia [3]. The main symptom of this disorder is chest pain or discomfort, which may radiate to the shoulder and arm. Typically, symptoms are exacerbated by physical exertion or emotional stress and decrease during rest [4].

CAD is the most common reason for a single cause of mortality and loss of disability-adjusted life years globally. In 2015, the CAD caused 8.9 million deaths and 164 million DALYs [5]. 

The main risk factors of CAD are type 2 diabetes mellitus [6], cigarette smoking [7], sedentary lifestyle [8], high cholesterol level [9], arterial hypertension [10], and obstructive sleep apnea [11]. 

Coronarography is the primary method used to diagnose significant coronary artery stenosis [12]. Its disadvantages are its cost and risk of vessel damage, and the physiological effect of the stenosis on myocardial function cannot be clearly assessed. To assess the physiological significance of coronary artery stenosis, the FFR (fractional flow reserve) is measured during coronary angiography [13]. During the measurement, it is necessary to insert a coronary pressure guidewire and administer a vasodilator. There is a low risk of damage to the coronary artery during the measurement [14]. However, the relevance of computed tomography angiography (CCTA), which may be used to assess significant coronary artery diseases, is growing [15]. CCTA is a non-invasive, fast, reliable, and reproducible method that assesses the coronary artery calcium (CAC) score based on the presence of calcium in the coronary artery [16]. CAC is a mathematically estimated, quantitative, unit-free parameter characterizing the amount of calcium within atherosclerotic plaques in coronary walls [17]. The following cut-off points were used to assess the risk of significant coronary artery disease: CACS = 0—no risk, CACS 1–10—minimal risk, CACS 11–100—low risk, CACS 101–400—moderate risk, and CACS > 400—high risk. According to recent guidelines, CCTA is increasingly recommended for screening in asymptomatic individuals to identify those at high risk of developing coronary artery disease and cardiac events, as well as for assessing coronary artery obstruction in symptomatic individuals [18].

Obstructive sleep apnea (OSA) is one of the most common respiratory disorders that is characterized by recurrent complete (apneas) and partial (hypopneas) upper airway collapse events [19]. The event can cause intermittent hypoxemia, autonomic fluctuation, and sleep fragmentation [20]. Snoring, apnea, and sleepiness are the main symptoms of OSA, although fatigue, breathlessness and choking, erectile dysfunction, concentration problems, and even insomnia have been reported in some patients [21]. Notably, 40–80% of patients with hypertension, heart failure, atrial fibrillation, and ischemic heart disease suffer from OSA [11]. Sympathetic activation, low-grade inflammation, oxidative stress, and endoplasmic reticulum stress are induced by intermittent hypoxia and play a role in cardiometabolic dysfunction [20]. Approximately 34% and 17% of middle-aged men and women suffer from OSA [22]; generally, approximately one billion people meet the criteria for OSA [23]. 

This study aimed to evaluate the coronary artery calcium score in patients with obstructive sleep apnea, specifically assessing the relationship between CACS and diagnosed OSA and between OSA severity and CACS.

## 2. Materials and Methods

A total of 124 patients were included in this study. The inclusion criteria included age > 18 years and coronary artery computed tomography indications and willingness to participate. Patients with previously diagnosed myocardial ischemic disease, chronic renal failure, history of stroke, and hyper- and hypothyroidism, and patients with insufficient coronary CT, severe mental disorders that prevent polysomnography, drug intake that can affect the breathing and/or neuromuscular activity, active malignancy, and active inflammation were excluded from the study.

All participants provided informed consent to participate in the study, and the study was approved by the Ethics Committee of Wroclaw Medical University (ID KB 369/2020) and conducted following the Declaration of Helsinki.

The clinical examination methodology included a medical history, measurement of total cholesterol, LDL cholesterol, HDL cholesterol, triglycerides, fasting glucose, and coronary computed tomography angiography. Full-night polysomnography was performed in patients with a high clinical probability of OSA. Blood pressure values were measured using the Korothov method. Hypertension and assessment of its degree were performed based on the European Society of Cardiology guidelines. Standard methods determined blood cholesterols, triglycerides, and glucose concentration according to the manufacturer’s instructions for the used reagent kits. The clinical characteristics of the studied group of patients are presented in Table 1.

Diagnosis of OSA was made based on the American Academy of Sleep Medicine (AASM) standards. Patients with a clinical probability of OSA were admitted to the Department of Internal Medicine, Occupational Diseases, Hypertension, and Clinical Oncology, where they underwent one-night polysomnography. Afterward, a certified polysomnographist assessed automatic 30 s epochs of polysomnograms, and the epochs were classified based on the standard criteria for sleep using the AASM 2013 Task Force. Respiratory events were documented as follows: no airflow (>90%) for ≥10 s was scored as apnea, while a ≥30% reduction in respiratory amplitude for ≥10 s with a ≥3% drop in blood oxygen saturation or arousal was scored as hypopnea. The total number of apneas and hypopneas per hour, defined as the AHI (apnea–hypopnea index) was used to assess the severity of OSA. Taking into account the value of the AHI, mild (5 ≤ AHI < 15), moderate (15 ≤ AHI < 30), and severe OSA (AHI ≥ 30) was diagnosed [24].

A 128-slice SOMATOM Definition Dual-Source CT scanner (Siemens Healthineers, Erlangen, Germany) was used to perform coronary computed tomography angiography. The study protocol included the following phases: topogram, a phase without an intravenous contrast agent to estimate the coronary artery calcium index (CACS), bolus tracking, nitroglycerin administration, and a phase with an intravenous contrast agent to assess the heart and coronary arteries properly. Iodine-based non-ionic contrast agent iomeprol (Iomeron 400, Bracco UK Ltd., Wycombe, UK) was administered intravenously using an automatic syringe through the ulnar fossa veins. The CACS was calculated using the syngo.CT CaScoring application (Siemens Healthineers, Erlangen, Germany). The software automatically classified any lesion ≥ 1 mm^2^ and density ≥ 130 Hounsfield units (HUs) as calcification. Each lesion classified as calcification was then classified as a lesion in the corresponding coronary arteries, namely the left main (LM), left anterior descending (LAD), left circumflex (LCX), and right coronary artery (RCA). Based on Agatston’s algorithm, the application calculated the CACS for each coronary artery as well as the total CACS. Two experienced radiologists verified the calculated values. The risk of significant coronary artery disease was determined based on the CACS value. The following criteria were used: CACS = 0, practically no risk of significant CAD; CACS 1–10, minimal risk of significant CAD; CACS 11–100, mild risk of significant CAD; CACS 101–400, moderate risk of significant CAD; and CACS ≥ 400, high risk of significant CAD. The result of each patient undergoing a CT scan of the coronary arteries was prepared using the Coronary Artery Calcium Data and Reporting System (CAC-DRS). This system indicates the result of the total calcium score, the calcium score of individual coronary arteries, and the number of vessels involved in the atherosclerotic process. Based on the result, the system provides suggestions for further management for the primary and secondary prevention of cardiovascular incidents [25].

Statistical analysis was performed using Dell Statistica 13 software (Dell Inc., Tulsa, OK, USA). Mean, median, interquartile range, and standard deviation were calculated for quantitative variables. The normal distribution of variables was verified using Lilliefors and Shapiro–Wilk tests. The quantitative independent variables with a normal distribution were analyzed using the *t*-test for independent variables. Variables with a non-normal distribution were analyzed using the Mann–Whitney U test for the quantitative independent variables. A chi-square test with the highest reliability was used for the analysis of independent quantitative variables. Correlation and regression analysis were conducted to determine the relationship between the study variables. Due to the lack of normal distribution of the analyzed variables, non-parametric Spearman correlation coefficients were determined. Backward stepwise multiple regression analysis was performed. In addition, accuracy was tested by proposing cut-off points for the tests estimated from receiver operating characteristic (ROC) curves. The level of statistical significance was set at *p* < 0.05.

## 3. Results

The patients included in this study were divided into a group of individuals with OSA (group A) and a group without diagnosed OSA (group B). The mean age was 59.12 ± 9.09 and 59.50 ± 10.74, respectively. The mean AHI index in the subgroup with OSA was 20.44 ± 13.22/h. The clinical characteristics are shown in Table 1.

Group A had a significantly higher CACS than group B (550.25 ± 817.76 vs. 92.59 ± 164.56, *p* < 0.05). The calcium score of individual coronary arteries (LM, LAD, LCX, and RCA) was significantly higher in the group of patients with OSA than in those without diagnosed OSA. Considering the cardiovascular risk estimated based on the CACS, there were statistically significant differences between groups A and B in the categories of no risk of significant coronary artery disease (0.0% vs. 51.6%) and a high risk of significant coronary artery disease (40.3% vs. 4.8%). Patients without OSA were significantly more likely to have no atherosclerotic lesions in the coronary arteries than those with OSA. All details can be found in Table 2.

In the next step, we checked whether there were statistically significant differences in the OSA group, considering disease severity as a differentiating factor. In group A, patients with severe OSA and moderate OSA had a significantly higher CACS than patients with mild OSA (910.04 ± 746.31, 833.35 ± 1129.87, 201.66 ± 192.04, *p* < 0.05). All the details are shown in Table 3.

**Figure 1:**
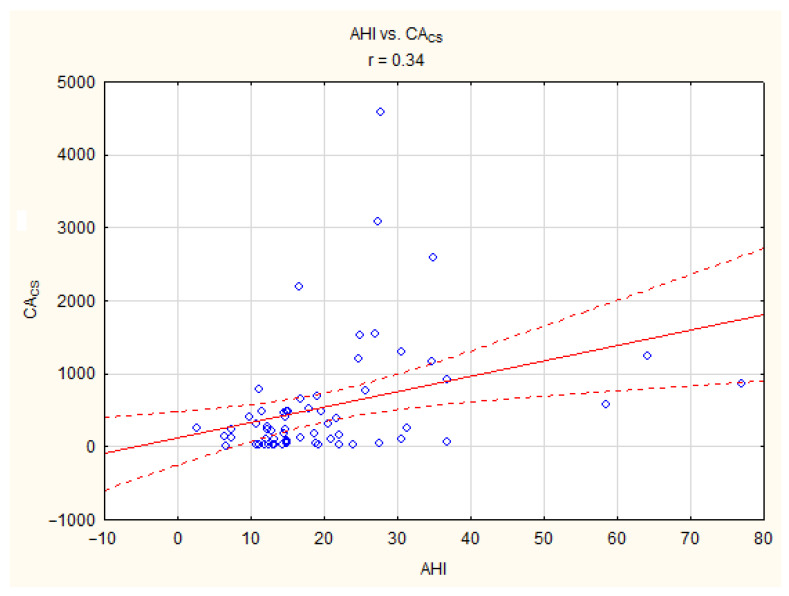
Linear relationship between AHI (/h) and CA_cs_ in patients with obstructive sleep apnea study group.

A statistically significant positive correlation was found between the AHI and CACS (r = 0.34, *p* < 0.05). Furthermore, a statistically significant correlation was observed between the CACS and age, BMI, and triglyceride levels. All details are presented in Table 4 and Figure 1.

To verify the independence of the obtained relationships between the basic body parameters (age, gender, and BMI), blood pressure (systolic and diastolic blood pressure), the basic biochemical parameters (total cholesterol concentration, triglycerides concentration, and glucose concentration), history of concomitant cardiovascular diseases (arterial hypertension, type 2 diabetes, hypercholesterolemia, and peripheral artery diseases), smoking, obstructive sleep apnea, and the CACS, backward stepwise multiple regression analysis was performed, obtaining the model presented in Table 5. The regression analysis showed that OSA, male gender, older age, type 2 diabetes, peripheral arterial disease, and smoking were independent risk factors for higher CACS values.

**Figure 2:**
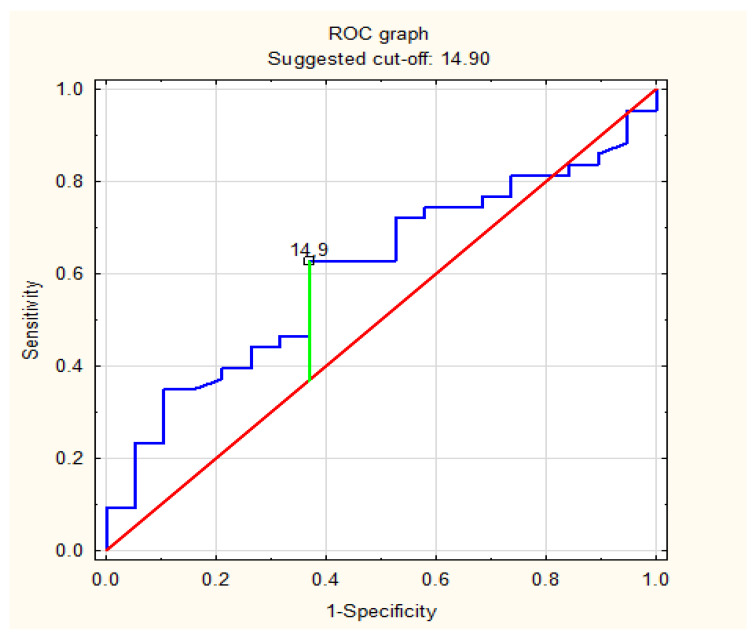
ROC curve of prediction of at least moderate risk of significant coronary artery disease based on AHI (/h) values in a group of patients with obstructive sleep apnea.

**Figure 3:**
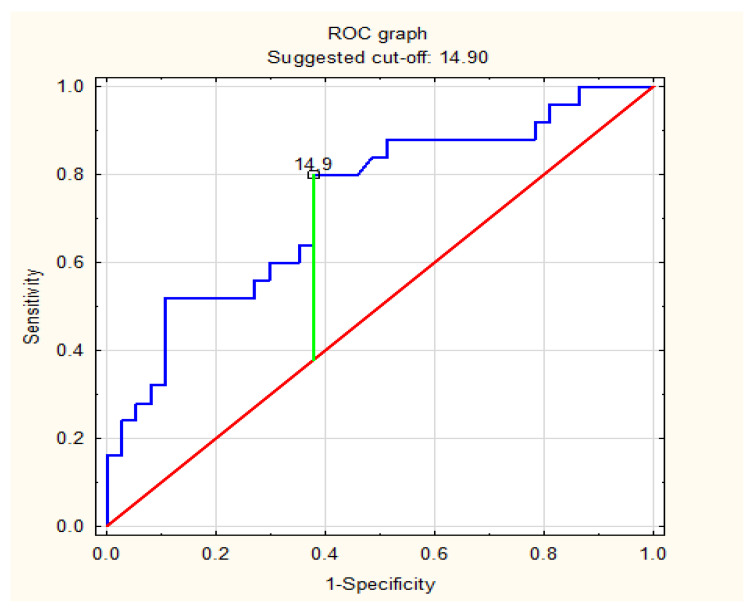
ROC curve of prediction of significant risk of significant coronary artery disease based on AHI (/h) values in a group of patients with obstructive sleep apnea.

ROC analysis identified the optimal AHI values that were predictive factors for a specific risk of significant CAD. Based on the ROC curve analysis, an AHI ≥ 14.9 was detected as a predictor of at least moderate risk of significant coronary artery disease with a sensitivity and specificity of 63.2% and 62.8%, respectively. AHI ≥ 14.9 predicted a high risk of significant coronary artery disease with a sensitivity and specificity of 62.2% and 80.0%, respectively. All details are presented in Table 6 and Figure 2 and Figure 3. 

## 4. Discussion

Patients who underwent coronary artery tomography were eligible for the study. Patients with a high probability of OSA underwent polysomnography. The results of this study showed that patients with OSA had significantly higher CACS than patients without OSA, in terms of both their global and individual coronary artery scores. Based on the CACS, the risk of significant CAD was calculated. Statistically significant differences between groups A and B were shown in patients with practically no risk and high risk of CAD. In the next step of the analysis, patients with diagnosed OSA were compared among themselves, considering the severity of the disease. In our study, group A patients with severe OSA and moderate OSA had statistically significantly higher CACS than patients with mild OSA. Moreover, a statistically significant positive correlation was found between the AHI and CACS in our group of patients. The regression analysis showed that OSA, male gender, older age, type 2 diabetes, peripheral arterial disease, and smoking were independent risk factors for higher CACS values. AHI ≥ 14.9 predicted a high risk of significant coronary artery disease.

There are few studies in the available literature on the association of OSA with the CACS. Sea et al., conducted a study involving 461 patients who underwent polysomnography and coronary artery tomography. Their analysis, after excluding the confounding factors, showed that lower saturation was independently associated with the CACS. There was no association of other parameters measured during polysomnography with the CACS [26]. In addition, in a subsequent study, the authors decided to assess the progression of subclinical CAD using the CACS. Patients underwent polysomnography and computed tomography of the coronary arteries. A follow-up CT scan was performed at any time. Total sleep time of SaO2 < 90%, the percentage of time of SaO2 < 90%, and the degree of mean oxygen desaturation were significantly correlated with CACS progression, even after excluding the confounding factors. The above study indicates that a lack of OSA treatment is associated with CAD progression [27].

Medeiros et al., evaluated the hypothesis of an association between OSA and the presence of atherosclerotic coronary lesions. They analyzed women aged 45–65 years without known cardiovascular disease. In contrast to our study, cardiovascular risk was not differentiated by the CACS value; CACS > 100 Agatston scores were used as the cut-off point. Based on the regression analysis, moderate or severe OSA was indicated as an independent risk factor for the presence of atherosclerotic lesions in the coronary arteries, which is consistent with the results of our study [28]. In contrast, in another study, Arik et al., showed that the AHI was weakly correlated with the CACS. In univariate analysis, age, AHI, basal oxygen desaturation, and oxygen desaturation index were associated with the CACS. However, in regression analysis, only the AHI and age were independent predictors of atherosclerotic lesions in the coronary arteries, which partly coincides with the results of our study, in which the regression analysis revealed that OSA, sex (male), type 2 diabetes mellitus, age, and smoking were independent predictors of significant CAD [29]. Bikov et al., investigated the association between OSA and CAD, showing that segment involvement and segment stenosis scores were higher in patients with OSA than in the control group. Furthermore, these indices significantly correlated with the severity of OSA. However, no significant correlation was shown between the CACS and OSA [30].

In a meta-analysis, Hao et al., showed an association between OSA and the presence of atherosclerotic lesions in patients without symptoms of heart disease. Furthermore, in a pairwise comparison, they indicated that the CACS might depend on the severity of OSA, which corresponds to our results [31].

CACS is a good method for detecting calcified atherosclerotic plaques and determining the risk of significant CAD. However, non-calcified plaques are identified in approximately 10% of patients with CACS = 0 [32]. A study is available in which the authors investigated the association between coronary non-calcified plaques and the severity of stenosis in patients with OSA. They showed that non-calcified plaques were significantly more common in patients with OSA than in patients without OSA. Patients with OSA also had more severe stenosis and a greater number of involved vessels than those without OSA. [33] Similar observations have also been reported in other studies [34,35,36,37]. Criqui et al., showed that higher coronary artery calcium density was associated with a lower cardiovascular disorder risk, affecting plaque stabilization [17].

Obstructive sleep apnea is a common sleep-breathing disorder affecting an increasing number of patients [38]. OSA is associated with a higher incidence of hypertension [39], coronary artery disease [5], stroke and cardiac arrhythmias [40], and type 2 diabetes mellitus [41]. The pathogenesis of the disease includes intermittent hypoxia, oxidative stress, and endothelial dysfunction, which are involved in the progression of the atherosclerotic process [20].

Coronary artery calcium is a highly specific feature of atherosclerosis in the coronary arteries, and the CACS is one of the best-studied and available tests in cardiovascular risk assessment. The development of CAC is understood as an active pathogenic process that can be stopped by controlling cardiovascular risk factors [42]. However, a different prevalence of coronary artery calcium was shown between Caucasians and the other three ethnic groups. In addition, it was revealed that the scores of all four ethnic groups with similar strength coronary artery calcium can be used to assess the probability of significant CAD [43].

The assessment of coronary artery calcium allows for the assessment of the likelihood of significant CAD and the implementation of primary prevention. CACS = 0 is the best predictive marker of practically no risk of significant CAD. In contrast, patients with CACS > 0, who are more likely to have significant CAD than having practically no risk, will benefit from pharmacotherapy [25]. However, CACS = 0 cannot be used alone to exclude significant CAD in patients with symptoms of CAD. In a study involving 2088 patients with symptoms of coronary artery disease, Kim et al., found CACS = 0 in 1114 patients, 48 of whom were diagnosed with significant CAD in the next step [44]. In another study, Aslan et al., revealed that age >50 years, male sex, and diabetes were independently associated with non-calcified coronary plaques, and in these patient groups, coronary computed tomography angiography is more recommended [45]. 

In our study, based on the ROC curve analysis, a cut-off point of AHI ≥ 14.9 was found to be a possible predictor of significant CAD. Therefore, a global assessment of the coronary arteries based on computed tomography angiography should be considered to exclude coronary artery disease even in asymptomatic patients with OSA. 

Obstructive sleep apnea is associated with CAD. It is important to select patients at risk based on the presence of the risk factors of OSA to perform an appropriate diagnosis and, if OSA is confirmed, to implement treatment as soon as possible. In patients with higher AHI values, it is advisable to diagnose CAD by evaluating the CACS, but, as shown in the study cited above, there is a risk that even with CACS = 0, CAD cannot be ruled out unequivocally. Therefore, contrast-enhanced CT angiography should be considered in this case to detect non-calcified atherosclerotic plaques.

The limitations of our study include the relatively small size of the groups and their heterogeneity. It should be noted, however, that the studied groups with and without OSA did not differ in the incidence of cardiovascular risk factors and the incidence of coexisting cardiovascular diseases. In addition, the regression analysis was used in our statistical analysis, which made it possible to assess the impact of the potential modifying factors on the assessed relationship between OSA vs. CACS. This approach was used to demonstrate the independence of this relationship from other co-occurring factors. The main methodology limitation of our study is the lack of polysomnography in patients at low risk of OSA. Unfortunately, even a low risk does not exclude OSA. Another major problem is the non-simultaneous performance of polysomnography and coronary computed tomography. Only the AHI was included in the analysis performed. As a next step, the relationship between hypoxemia, sleep fragmentation, arousal, and CAD severity would have to be investigated. A review of the literature showed that the above parameters have a particular association with oxidative stress, inflammation, and endothelial dysfunction, which may be related to atherosclerosis. Other parameters available in polysomnography were not included, which requires further research. In addition, the relationship between the use of appropriate treatment and the severity of atherosclerotic lesions, as assessed with the CACS, should be investigated in the next step. In our study, we investigated the association of OSA with the CACS, so we did not check the association of OSA with non-calcified atherosclerotic plaques, which also needs to be explored in the future. Other quantitative parameters of cardiac morphology and function, which can be assessed in coronary computed tomography angiography images, should also be verified in terms of their dependence on the occurrence and severity of OSA.

## 5. Conclusions

1. Obstructive sleep apnea should be considered an independent predictor of a high risk of significant coronary artery disease (based on the coronary artery calcium score);
2. OSA, male gender, older age, type 2 diabetes, peripheral arterial disease, and smoking should be considered as independent risk factors for higher CACS values;
3. AHI ≥ 14.9 was detected as a potential predictor of at least a moderate risk of significant coronary artery disease;
4. The CACS may depend on the severity of OSA.

## Figures and Tables

**Table 1:** Clinical characteristics of the study groups: group A—patients with obstructive sleep apnea; group B—patients without obstructive sleep apnea.

	Group A (n = 62)	Group B (n = 62)	*p*
Age (years)	59.12 ± 9.09	59.50 ± 10.74	ns
BMI (kg/m^2^)	31.09 ± 3.05	24.21 ± 3.46	0.001
overweight (%)	35.5	27.4	ns
obesity (%)	62.9	6.4	0.001
Gender (%)			
men	40.3	43.5	ns
women	59.7	56.5	
Arterial hypertension (%)	56.4	53.2	ns
sBP (mmHg)	142.66 ± 7.98	144.52 ± 7.50	ns
dBP (mmHg)	88.39 ± 5.56	88.63 ± 5.52	ns
Diabetes mellitus type 2 (%)	21.0	19.3	ns
Glucose (mg/dL)	111.53 ± 36.47	120.71 ± 42.43	ns
Hypercholesterolemia (%)	66.1	56.4	ns
Lower-limb arteriosclerosis (%)	21.0	22.6	ns
Total cholesterol (mg/dL)	198.35 ± 41.06	203.95 ± 44.08	ns
Triglycerides (mg/dL)	190.13 ± 123.32	173.44 ± 83.60	ns
Cigarette smoking (%)	30.6	37.1	ns
Obstructive sleep apnea (%) Mild Moderate Severe	100.0 46.8 37.1 16.1	0.0 0.0 0.0 0.0	-
AHI (/h)	20.44 ± 13.22	-	-

AHI—apnea–hypopnea index; BMI—body mass index; dBP—diastolic blood pressure; sBP—systolic blood pressure; ns—non-significant.

**Table 2:** Coronary artery calcification score parameters in the study groups: group A—patients with obstructive sleep apnea; group B—patients without obstructive sleep apnea.

	Group A (n = 62)	Group B (n = 62)	*p*
CA_CS_	550.25 ± 817.76	92.59 ± 164.56	0.001
LM_CS_	31.37 ± 85.62	1.06 ± 4.95	0.006
LAD_CS_	235.25 ± 244.77	57.38 ± 102.22	0.001
LCX_CS_	84.97 ± 201.75	11.51 ± 30.58	0.005
RCA_CS_	196.98 ± 512.29	22.61 ± 71.34	0.009
Risk of significant CAD			
practically no risk	0.0	51.6	0.001
minimal	1.6	11.3	ns
mild risk	29.0	8.1	ns
moderate	29.0	24.2	ns
high	40.3	4.8	0.001
CAC-DRS A			
A0	0.0	53.2	0.001
A1	30.6	17.7	ns
A2	24.2	21.0	ns
A3	45.2	8.1	0.001
CAC-DRS N			
N0	0.0	53.2	0.001
N1	21.0	12.9	ns
N2	14.5	8.1	ns
N3	37.1	22.6	ns
N4	27.4	3.2	0.001

CACS—coronary artery calcium score; CAC-DRS A—Agatston scoring in Coronary Artery Calcium Data and Reporting System; CAC-DRS N—number of vessels in Coronary Artery Calcium Data and Reporting System CAD—coronary artery disease; LAD_CS_—left anterior descending calcium score; LCX_CS_—left circumflex calcium score; LM_CS_—left main calcium score; RCA_CS_—right coronary artery calcium score; ns—non-significant.

**Table 3:** Coronary artery calcium score assessment in the study subgroups: subgroup A1—patients with mild obstructive sleep apnea; subgroup A2—patients with moderate obstructive sleep apnea; subgroup A3—patients with severe obstructive sleep apnea.

	Subgroup A1 (n = 29)	Subgroup A2 (n = 23)	Subgroup A3 (n = 10)	*p*
CA_CS_	201.66 ± 192.04	833.35 ± 1129.87	910.04 ± 746.31	A1-A2: 0.004 A1-A3: 0.014
LM_CS_	9.58 ± 21.79	49.74 ± 128.84	52.32 ± 68.94	ns
LAD_CS_	124.58 ± 114.79	308.30 ± 294.83	388.18 ± 271.09	A1-A2: 0.004 A1-A3: 0.002
LCX_CS_	24.49 ± 38.90	166.20 ± 309.02	73.58 ± 94.57	ns
RCA_CS_	42.21 ± 81.15	305.38 ± 737.27	396.46 ± 527.36	ns

CA_CS_—coronary artery calcium score; LAD_CS_—left anterior descending calcium score; LCX_CS_—left circumflex calcium score; LM_CS_—left main calcium score; RCA_CS_—right coronary artery calcium score; ns—non-significant.

**Table 4:** Results of correlation analysis in the study group of patients.

	CA_CS_	
	r	*p*
Age (years)	0.40	0.001
BMI (kg/m^2^)	0.31	0.001
sBP (mmhg)	−0.03	ns
dBP (mmhg)	−0.01	ns
Glucose (mg/dL)	0.09	ns
Total cholesterol (mg/dL)	0.08	ns
Triglycerides (mg/dL)	0.23	0.008
AHI (/h)	0.34	0.006

AHI—apnea–hypopnea index; BMI—body mass index; CA_CS_—coronary artery calcium score; dBP—diastolic blood pressure; r—correlation coefficient; sBP—systolic blood pressure; ns—non-significant.

**Table 5:** Backward stepwise multiple regression model for the dependent variable CACS.

Model for: CA_cs_			
	Regression Coefficient	SEM of RC	*p*
Intercept	−159.972	62.463	0.047
Men	180.852	86.480	0.042
Age (years)	10.473	4.941	0.037
Type 2 diabetes mellitus	253.977	111.207	0.024
Peripheral arterial disease	453.292	112.049	0.001
Smoking	337.100	111.359	0.003
Obstructive sleep apnea	241.330	107.825	0.027

CA_cs_—coronary artery calcium score; SEM of RC—standard error of the mean of the regression coefficient.

**Table 6:** Results of sensitivity and specificity analysis of AHI as a predictor of the risk of significant coronary artery disease assessed with coronary artery calcium score values in a group of patients with obstructive sleep apnea.

	Prediction of ≥ Moderate Risk of Significant Coronary Artery Disease	Prediction of Significant Risk of Significant Coronary Artery Disease
AHI (/h) cut-off for predicting the risk of significant coronary artery disease	≥14.9	≥14.9
Sensitivity	0.632	0.622
Specificity	0.628	0.800
Accuracy	0.629	0.694
Positive predictive values	0.429	0.821
Negative predictive values	0.794	0.588
Likelihood ratios (positive)	1.697	3.108
Likelihood ratios (negative)	0.587	0.473

## Data Availability

The data presented in this study are available on request from the corresponding author.
